# Ethical arguments concerning human-animal chimera research: a systematic review

**DOI:** 10.1186/s12910-020-00465-7

**Published:** 2020-03-23

**Authors:** Koko Kwisda, Lucie White, Dietmar Hübner

**Affiliations:** 1 CELLS – Centre for Ethics and Law in the Life Sciences, Leibniz University Hannover, Otto-Brenner-Strasse 1, 30159 Hannover, Germany; 2 Institute of Philosophy, Leibniz University Hannover, Im Moore 21, 30167 Hannover, Germany

**Keywords:** Human-animal chimeras, Chimera research, Ethics, Systematic review

## Abstract

**Background:**

The burgeoning field of biomedical research involving the mixture of human and animal materials has attracted significant ethical controversy. Due to the many dimensions of potential ethical conflict involved in this type of research, and the wide variety of research projects under discussion, it is difficult to obtain an overview of the ethical debate. This paper attempts to remedy this by providing a systematic review of ethical reasons in academic publications on human-animal chimera research.

**Methods:**

We conducted a systematic review of the ethical literature concerning human-animal chimeras based on the research question: “What ethical reasons have been given for or against conducting human-animal chimera research, and how have these reasons been treated in the ongoing debate?” Our search extends until the end of the year 2017, including MEDLINE, Embase, PhilPapers and EthxWeb databases, restricted to peer-reviewed journal publications in English. Papers containing ethical reasons were analyzed, and the reasons were coded according to whether they were endorsed, mentioned or rejected.

**Results:**

Four hundred thirty-one articles were retrieved by our search, and 88 were ultimately included and analyzed. Within these articles, we found 464 passages containing reasons for and against conducting human-animal chimera research. We classified these reasons into five categories and, within these, identified 12 broad and 31 narrow reason types.

15% of the retrieved passages contained reasons in favor of conducting chimera research (Category P), while 85% of the passages contained reasons against it. The reasons against conducting chimera research fell into four further categories: reasons concerning the creation of a chimera (Category A), its treatment (Category B), reasons referring to metaphysical or social issues resulting from its existence (Category C) and to potential downstream effects of chimera research (Category D). A significant proportion of identified passages (46%) fell under Category C.

**Conclusions:**

We hope that our results, in revealing the conceptual and argumentative structure of the debate and highlighting some its most notable tendencies and prominent positions, will facilitate continued discussion and provide a basis for the development of relevant policy and legislation.

## Background

Research involving the mixture of human and animal materials has been controversial from its inception. Proposed research projects, particularly geared towards the production of human organs for transplantation in an animal host [1–7], involving the implantation of human brain stem cells into other animals [8–10], or aiming at the creation of human-animal admixed embryos [11–14], have spurred this debate, generating a wide spectrum of arguments both for and against such research in public and academic discourse. The far-reaching nature of these controversies, involving a large variety of factors debated over a range of different venues, makes it particularly difficult, and important, to obtain a general overview of the debate.

This paper provides a systematic review of ethical arguments contained in academic publications on human-animal chimera^1^ research. Systematic reviews involve searching databases in a methodical and reproducible way, retrieving literature according to predefined inclusion criteria, and analyzing this literature in order to answer a specific research question. Originally a tool of the social sciences, their use was extended to medical contexts, providing comprehensive information on research findings and clinical results in order to facilitate decision-making. More recently, this method has been extended to philosophical bioethics, taking as its focus the argument-based literature found in this field [15, 16].

The research question underlying this systematic review is: *“What ethical reasons have been given for or against conducting human-animal chimera research, and how have these reasons been treated in the ongoing debate?”* In order to adequately answer this question, we produced a detailed, multilayered classification system of reasons, which elucidates the basic conceptual structure of the debate. We provide quantitative information on how often reasons have been endorsed, rejected, or merely mentioned, to give a thorough account of positions, tendencies and camps within the literature. Finally, we comment on the nature of our findings in the discussion section, giving an indication of the factors that might explain certain notable patterns in the results. By providing structure to the debate, drawing attention to central concerns, and uncovering certain specific features of the current dispute, including potential argumentative gaps and straw man arguments, we aim to establish a basis for continued discussion and to facilitate the development of relevant policy and legislation.

## Methods

### Literature search and eligibility criteria

**Fig. 1:**
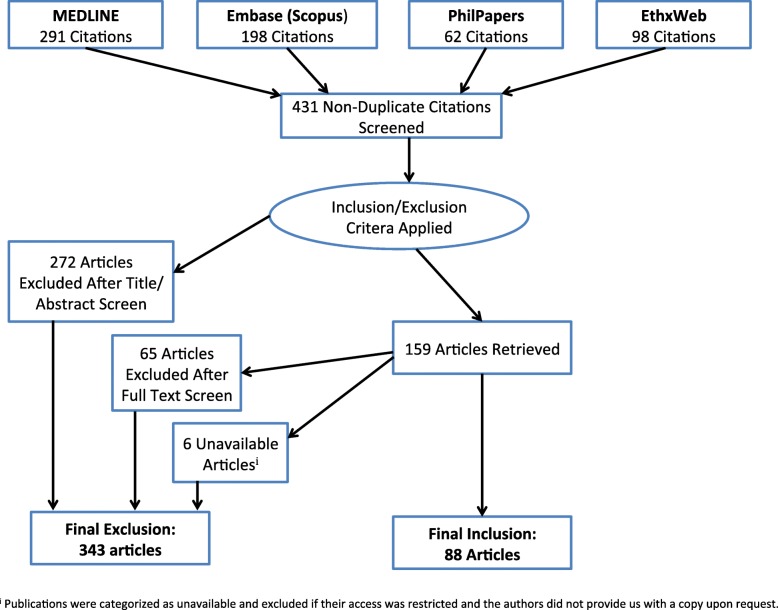
Flowchart documenting the retrieval of publications, the application of inclusion/exclusion criteria and further exclusions after full text screen

To minimize potential bias and ensure an exhaustive retrieval, several databases were screened, namely MEDLINE, Embase, PhilPapers and EthxWeb (see Fig. 1).

Databases were searched up to 31 December 2017. Database-specific controlled vocabulary and search strings applied are summarized in Table 1. Respective search results were fused with a bibliography software (Thomson Reuters EndNote®) and duplicate references removed. All 88 included publications are listed alphabetically in Table 2.

**Table 1:** List of databases screened with respective search strings used

Database	Search string^a^
MEDLINE	((((“Chimera”[Mesh])) OR (chimera))) AND ((((((“Ethics”[Mesh])) OR (ethics)) OR (ethical)) OR (bioethics)) OR (bioethical))
Embase (Scopus)	Bioethic* OR ethic* AND chimer* (restricted to “Articles” and “Articles in Press”)
PhilPapers	(ethic* bioeth*) (chimer*) Fuzzy filter advanced
EthxWeb	Chimer+ AND (ethic+ OR bioethic+) restricted to journal articles
Updates via email update on various databases	As above

^a^Words refer to controlled vocabulary of respective databases

**Table 2:** Included and analyzed publications in alphabetical order

[17]	Abelman M, O’Rourke PP, Sonntag KC (2012) Part-human animal research: the imperative to move beyond a philosophical debate. Am J Bioeth **12** (9):26–8
[18]	Ankeny RA (2003) No real categories, only chimeras and illusions: the interplay between morality and science in debates over embryonic chimeras. Am J Bioeth **3** (3):31–3
[19]	Anton R (2016) On recent advances in human engineering Provocative trends in embryology, genetics, and regenerative medicine. Politics Life Sci **35** (2):54–68
[20]	Austriaco NP (2006) How to navigate boundaries: a reply to The American Journal of Bioethics. Natl Cathol Bioeth Q **6** (1):61–71
[21]	Badura-Lotter G, Fangerau H (2014) Human-animal chimeras: not only cell origin matters. Am J Bioeth **14** (2):21–2
[12]	Bahadur G, Iqbal M, Malik S et al. (2008) Admixed human embryos and stem cells: legislative, ethical and scientific advances. Reprod Biomed Online **17** (Suppl 1):25–32
[22]	Baylis F (2008) Animal eggs for stem cell research: a path not worth taking. Am J Bioeth **8** (12):18–32
[8]	Baylis F, Fenton, A. (2007) Chimera research and stem cell therapies for human neurodegenerative disorders. Camb Q Healthc Ethics **16** (2):195–208
[23]	Baylis F, Robert JS (2007) Part-human chimeras: worrying the facts, probing the ethics. Am J Bioeth **7** (5):41–5
[24]	Benham B, Haber M (2008) Moral confusion and developmental essentialism in part-human hybrid research. Am J Bioeth **8** (12):42–4
[25]	Bok H (2003) What’s wrong with confusion? Am J Bioeth **3** (3):25–6
[4]	Bourret R, Martinez E, Vialla F et al. (2016) Human-animal chimeras: ethical issues about farming chimeric animals bearing human organs. Stem Cell Res Ther **7** (1):87
[26]	Cabrera Trujillo LY, Engel-Glatter S (2014) Human-Animal Chimera: A Neuro Driven Discussion? Comparison of Three Leading European Research Countries. Sci Eng Ethics
[13]	Camporesi S, Boniolo G (2008) Fearing a non-existing Minotaur? The ethical challenges of research on cytoplasmic hybrid embryos. J Med Ethics **34** (11):821–5
[27]	Capps B (2017) Do Chimeras Have Minds? Camb Q Healthc Ethics **26** (4):577–591
[28]	Castle D (2003) Hopes against hopeful monsters. Am J Bioeth **3** (3):28–30
[29]	Chan S (2014) Hidden anthropocentrism and the “benefit of the doubt”: problems with the “origins” approach to moral status. Am J Bioeth **14** (2):18–20
[30]	Chapman A, Hiskes AL (2008) Unscrambling the eggs: cybrid research through an Embryonic Stem Cell Research Oversight Committee (ESCRO) lens. Am J Bioeth **8** (12):44–6
[31]	Charland LC (2003) Are there answers? Am J Bioeth **3** (3):1–2
[32]	Cheshire WP, Jr. (2007) The moral musings of a murine chimera. Am J Bioeth **7** (5):49–50
[33]	Cohen CB (2003) Creating human-nonhuman chimeras: of mice and men. Am J Bioeth **3** (3):3–5
[34]	Cooley DR (2008) Genetically Engineering Human-Animal Chimeras and Lives Worth Living. Between The Species **8**:1–19
[35]	Coors ME (2006) Considering chimeras: the confluence of genetic engineering and ethics. Natl Cathol Bioeth Q **6** (1):75–87
[36]	de Melo-Martin I (2008) Chimeras and human dignity. Kennedy Inst Ethics J **18** (4):331–46
[37]	deGrazia D (2007) Human-animal chimeras: human dignity, moral status, and species prejudice. Metaphilosophy **38** (2–3):310–329
[38]	DiSilvestro R (2012) The two-essence problem that wasn’t. Am J Bioeth **12** (9):34–5
[39]	Eberl JT (2007) Creating non-human persons: might it be worth the risk? Am J Bioeth **7** (5):52–4
[40]	Eberl JT (2012) Ontological kinds versus biological species. Am J Bioeth **12** (9):32–4
[41]	Eberl JT, Ballard RA (2008) Exercising restraint in the creation of animal-human chimeras. Am J Bioeth **8** (6):45–6
[42]	Eberl JT, Ballard RA (2009) Metaphysical and ethical perspectives on creating animal-human chimeras. J Med Philos **34** (5):470–86
[43]	Franklin S (2003) Drawing the line at not-fully-human: what we already know. Am J Bioeth **3** (3):25–27
[44]	Gerrek ML (2008) Who really causes the lady to vanish? Am J Bioeth **8** (12):46–7
[45]	Greely HT (2003) Defining chimeras...and chimeric concerns. Am J Bioeth **3** (3):17–20
[9]	Greely HT, Cho MK, Hogle LF et al. (2007) Thinking about the human neuron mouse. Am J Bioeth **7** (5):27–40
[46]	Greene M, Schill K, Takahashi S et al. (2005) Ethics: Moral issues of human-non-human primate neural grafting. Science **309** (5733):385–6
[47]	Haber MH, Benham B (2012) Reframing the ethical issues in part-human animal research: the unbearable ontology of inexorable moral confusion. Am J Bioeth **12** (9):17–25
[48]	Heathcotte B, Robert JS (2006) The strange case of the humanzee patent quest. Natl Cathol Bioeth Q **6** (1):51–9
[49]	Hermeren G (2015) Ethical considerations in chimera research. Development **142** (1):3–5
[50]	Hyun I (2015) From naive pluripotency to chimeras: a new ethical challenge? Development **142** (1):6–8
[51]	Hyun I (2016) What’s Wrong with Human/Nonhuman Chimera Research? PLoS Biol **14** (8):e1002535
[52]	Irvine R, Degeling C, Kerridge I (2012) Uncanny animals: thinking differently about ethics and the animal-human relationship. Am J Bioeth **12** (9):30–2
[53]	Johnston J, Eliot C (2003) Chimeras and “human dignity”. Am J Bioeth **3** (3):W6-w8
[14]	Jones DA (2010) Is the creation of admixed embryos “an offense against human dignity”? Hum Reprod Genet Ethics **16** (1):87–114
[54]	Jones DA (2012) The ethics of creating chimeras and other admixed organisms. Ethics and Medicine **28** (3):81–93
[55]	Karpowicz P (2003) In defense of stem cell chimeras: a response to “Crossing species boundaries”. Am J Bioeth **3** (3):17–19
[11]	Karpowicz P, Cohen CB, van der Kooy D (2004) It is ethical to transplant human stem cells into nonhuman embryos. Nat Med **10** (4):331–5
[56]	Karpowicz P, Cohen CB, van der Kooy D (2005) Developing human-nonhuman chimeras in human stem cell research: ethical issues and boundaries. Kennedy Inst Ethics J **15** (2):107–34
[57]	Knoppers BMJ, Yann (2007) Our social genome? Trends Biotechnol **25** (7):284–288
[58]	Kobayashi NR (2003) A scientist crossing a boundary: a step into the bioethical issues surrounding stem cell research. Am J Bioeth **3** (3):15–16
[59]	Lavieri RR (2007) The ethical mouse: be not like Icarus. Am J Bioeth **7** (5):57–8
[60]	Levine S, Grabel L (2017) The contribution of human/non-human animal chimeras to stem cell research. Stem Cell Res **24**:128–134
[61]	Masaki H, Nakauchi H (2017) Interspecies chimeras for human stem cell research. Development **144** (14):2544–2547
[62]	McGee DB (2003) Moral ambiguity? Yes. Moral confusion? No. Am J Bioeth **3** (3):11–12
[63]	Mirkes R (2006) Is it ethical to generate human-animal chimeras? Natl Cathol Bioeth Q **6** (1):109–30
[6]	Munsie M, Hyun I, Sugarman J (2017) Ethical issues in human organoid and gastruloid research. Development **144** (6):942–945
[64]	Murphy TF (2008) When is an objection to hybrid stem cell research a moral objection? Am J Bioeth **8** (12):47–9
[65]	Nelson JL (2008) Respecting boundaries, disparaging values. Am J Bioeth **8** (12):33–4
[66]	Palacios-Gonzalez C (2015) Ethical aspects of creating human-nonhuman chimeras capable of human gamete production and human pregnancy. Monash Bioeth Rev. **33** (2–3):181–202
[5]	Palacios-Gonzalez C (2016) The ethics of killing human/great-ape chimeras for their organs: a reply to Shaw et al. Med Health Care Philos **19** (2):215–25
[67]	Palacios-Gonzalez C (2017) Chimeras intended for human gamete production: an ethical alternative? Reprod Biomed Online **35** (4):387–390
[68]	Palacios-González C (2015) Human dignity and the creation of human–nonhuman chimeras. Medicine, Health Care and Philosophy **18** (4):487–499
[69]	Piotrowska M (2012) Who are my parents? Why assigning moral categories to genealogical relations leads to more confusion. Am J Bioeth **12** (9):28–30
[70]	Pusch AF (2015) Splices: When Science Catches Up with Science Fiction. NanoEthics **9** (1):55–73
[71]	Ravelingien A, Braeckman, J., Legge, M. (2006) On the moral status of humanized chimeras and the concept of human dignity. Between the Species **6**:1–22
[72]	Robert JS (2006) The science and ethics of making part-human animals in stem cell biology. Faseb j **20** (7):838–45
[73]	Robert JS, Baylis F (2003) Crossing species boundaries. Am J Bioeth **3** (3):1–13
[74]	Robert JS, Baylis F (2003) A response to commentators on “Crossing species boundaries”. Am J Bioeth **3** (3):W-c6
[75]	Robertson JA (2003) A response to “Crossing species boundaries” by Jason Scott Robert and Francoise Baylis. Am J Bioeth **3** (3):W-c5
[76]	Rollin BE (2007) Of mice and men. Am J Bioeth **7** (5):55–7
[77]	Rollin BE (2007) On chimeras. Zygon **42** (3):643–648
[78]	Sagoff M (2003) Transgenic chimeras. Am J Bioeth **3** (3):30–1
[10]	Sagoff M (2007) Further thoughts about the human neuron mouse. Am J Bioeth **7** (5):51–2
[79]	Salter B, Harvey A (2014) Creating problems in the governance of science: Bioethics and human/animal chimeras. Science and Public Policy **41** (5):685–696
[80]	Saniotis A (2013) Remaking homo: Ethical issues on future human enhancement. Ethics in Science and Environmental Politics **13** (1):15–21
[81]	Savulescu J (2003) Human-animal transgenesis and chimeras might be an expression of our humanity. Am J Bioeth **3** (3):22–5
[82]	Savulescu J, Skene L (2008) The kingdom of genes: why genes from animals and plants will make better humans. Am J Bioeth **8** (12):35–8
[83]	Schaub DJ (2006) Chimeras: from poetry to science. Natl Cathol Bioeth Q **6** (1):29–35
[84]	Seyfer TL (2006) An overview of chimeras and hybrids. Natl Cathol Bioeth Q **6** (1):37–49
[2]	Shaw D, Dondorp W, de Wert G (2014) Using non-human primates to benefit humans: research and organ transplantation. Medicine, Health Care and Philosophy **17** (4):573–578
[85]	Shaw D, Dondorp W, Geijsen N et al. (2014) Creating human organs in chimaera pigs: an ethical source of immunocompatible organs? J Med Ethics
[86]	Siegel AW (2003) The moral insignificance of crossing species boundaries. Am J Bioeth **3** (3):33–4
[87]	Streiffer R (2003) In defense of the moral relevance of species boundaries. Am J Bioeth **3** (3):37–8
[88]	Streiffer R (2005) At the edge of humanity: human stem cells, chimeras, and moral status. Kennedy Inst Ethics J **15** (4):347–70
[89]	Streiffer R (2010) Chimeras, moral status, and public policy: implications of the abortion debate for public policy on human/nonhuman chimera research. J Law Med Ethics **38** (2):238–50
[90]	Thompson PB (2003) Crossing species boundaries is even more controversial than you think. Am J Bioeth **3** (3):14–5
[91]	Urie KA, Stanley A, Friedman JD (2003) The humane imperative: a moral opportunity. Am J Bioeth **3** (3):20–21
[92]	Watt H (2007) Embryos and pseudoembryos: parthenotes, reprogrammed oocytes and headless clones Journal of Medical Ethics **33** (9):554–556
[93]	Zwanziger LL (2003) Crossing perspectival chasms about species. Am J Bioeth **3** (3):9–10

We restricted our search to English literature, due to the proficiencies of the authors and the availability of sources. We also focused exclusively on original, academic publications in international, peer-reviewed journals, excluding reports, surveys, encyclopedia entries, handbook articles, guidelines, opinions, editorials, reviews, monographs, anthologies, letters, web-posts and newspaper articles.

A publication was included only if it addressed at least one ethical reason concerning why human-animal chimera research should or should not be pursued.^2^ Decisions concerning whether articles should be included were based on the publications’ abstracts, or, if these were inconclusive, on a close reading of the full text. All 88 included publications are listed in Table 2.

### Extraction and categorization of reasons

For the development of the coding system for reasons, we followed the methodology suggested by Strech and Sofaer (2012) [15]. To adequately mirror the ongoing discussion and provide in-depth analysis, we distinguished between three stances taken regarding a reason:

- Mere mentioning of a reason (i.e., reiteration or consideration of a reason without unequivocal rejection or endorsement). This includes statements such as “X constitutes a reason against chimera research unless measures ABC are taken”, or “X does not constitute a reason against chimera research as long as measures ABC are taken”.
- Rejection of a reason.
- Endorsement of a reason OR development of own reason.

We coded each reason once per publication. For instance, if a reason was first mentioned but then ultimately rejected, this was only counted once as a rejection. Alternatively, if a reason was, for example, rejected multiple times within the same paper, perhaps on different grounds, only one passage was coded as a rejection. Note that an author may endorse a certain reason for one type of chimera, but, in the same article or in another publication, reject this very same reason with regard to another type of chimera.

In our categorization of types of reasons, we differentiated between broad types (e.g. A.2 “Human beings/human material might be mistreated/misused”) and narrow types (e.g. A.2.i “Human embryo protection may be neglected”, or A.2.ii “Undue forms of human egg donation may occur”), with each narrow type falling under one broad type. Each broad reason type, in turn, was collected under one of five main reason categories (see below).

The extraction and categorization of reasons unavoidably involves interpretation. To produce a stable coding system and ensure intercoder reliability we employed the following procedure: The publications that at initial inspection appeared to be more detailed and comprehensive were grouped together in a first cluster of seven publications. Two authors (D.H. and K.K.) identified and initially categorized text passages independently in this subsample, then discussed whether these passages displayed a reason and how it should be categorized. The result was a first version of the coding system. A second cluster of 20 publications, which still appeared to be relatively comprehensive, was then used to check theoretical saturation of the categorical spectrum, and to revise and fine-tune the categorization of reasons. At this point, the main categories and broad reason types had been established; only minor adjustments within the narrow types of reasons were subsequently necessary. All three authors (K.K., D.H., L.W.) then checked the extraction and categorization of reasons in a random sample of another five publications. Our assignment of reasons was largely consistent, which we took to demonstrate the validity of our category system.

Within the complete set of included articles, each publication was analyzed by at least two authors. In the event of any coding incongruities, concordance was reached through in-depth discussion.

## Results

### Publication characteristics

Our literature research retrieved 431 non-duplicate references, 88 of which were included (see Fig. 1). All articles were published between 2003 and 2017 in peer-reviewed journals. Table 3 characterizes the disciplines of the journals in which the articles were published.

**Table 3:** Journal disciplines for all included publications

Journal Disciplines	
Bioethics	46 (52.3%)
Science/Medicine	14 (15.9%)
Medical Ethics	8 (9.1%)
Theology	7 (7.9%)
Ethics General	5 (5.7%)
Ethics of Science/Technology	3 (3.4%)
Philosophy of Medicine	2 (2.3%)
Philosophy General	1 (1.1%)
Law	1 (1.1%)
Politics and Life Sciences	1 (1.1%)
**Total**	**88 (100%)**

### Categories, types and frequencies of reasons

Within the 88 retrieved publications, we found 464 text passages containing reasons. The latter fall into five main categories, 12 broad types, and 31 narrow types of reasons. Tables documenting the frequency of reason types for each category can be found below. A quotation exemplifying each reason type is contained in Additional file 1.

Of the five main categories, *Category P* (*positive reasons)* contains discussion of reasons in favor of chimera research*.* This category contains 15% of all identified passages (70 passages), making it the third most debated category. The reasons within Category P were divided into four broad reason types: creating chimeras might lead to advances in basic research (P.1), produce benefits for humans (P.2), prevent direct harm to humans or animals (P.3), or entail other benefits (P.4). 31% of all coded passages in this category are mentions, 7% are rejections, and 61% are endorsements (see Table 4).

**Table 4:** Mentions, rejections and endorsements of positive reasons (Category P)

Positive Reasons (Category P)	
P.1: The creation of chimeras may advance basic research	*Mention* [23, 35, 54, 71, 79] *Reject* [8, 72] *Endorse* [9, 12, 13, 17, 41, 42, 59–61, 75, 76, 81, 82]
P.2: The creation of chimeras may produce benefits for humans	
P.2.i: New therapies might be developed on the basis of chimera research	*Mention* [8, 20, 41, 43, 54, 66, 71, 79, 84] *Reject* [11, 22, 23] *Endorse* [2, 9, 12, 13, 17, 34, 35, 39, 42, 55, 60, 67, 72, 75, 85]
P.2.ii: Chimeras might serve as sources of transplantable organs and tissues	*Mention* [5, 63] *Reject* [none] *Endorse* [2, 4, 34, 41, 42, 51, 60, 66, 72, 85]
P.2.iii: Chimera research might open ways to human enhancement	*Mention* [80] *Reject* [none] *Endorse* [81]
P.3: The creation of chimeras may prevent direct harm to humans or animals^a^	*Mention* [12, 23, 66] *Reject* [none] *Endorse* [13, 41, 42]
P.4: The creation of chimeras may have other benefits^b^	*Mention* [46, 80] *Reject* [none] *Endorse* [13]

^a^ E.g. by helping to replace human subjects or laboratory animals in biomedical research

^b^ E.g. by fostering the preservation of endangered species, or by allowing animal enhancement

The remaining four categories contain discussions of reasons against chimera research.

*Category A* (*chimera creation)* contains reasons pertaining to the process leading to the creation of a chimera. 11% of all identified passages (53 passages) are in this category, making it the second-least debated category. There are two concerns here, reflected by the two broad reason types: the potential mistreatment of animals (A.1), and the potential mistreatment of human beings or misuse of human material (A.2). 58% of all coded passages in this category are mentions, 28% are rejections, and 13% are endorsements (see Table 5).

**Table 5:** Mentions, rejections and endorsements of reasons concerning chimera creation (Category A)

Reasons Concerning Chimera Creation (Category A)	
A.1: Animals might be mistreated	
A.1.i: General animal welfare may be infringed	*Mention* [9, 14, 22, 26, 49, 54, 59, 65, 80, 89] *Reject* [76, 79] *Endorse* [37]
A.1.ii: Special protection of higher animals such as primates may be infringed	*Mention* [22, 26, 46] *Reject* [2] *Endorse* [37, 42]
A.2: Human beings/human material might be mistreated/misused	
A.2.i: Human embryo protection may be neglected	*Mention* [12, 14, 24, 26, 44, 49, 59, 64, 65, 79, 89] *Reject* [9, 66, 76] *Endorse* [63, 92]
A.2.ii: Undue forms of human egg donation may occur	*Mention* [12, 14, 24, 54, 65] *Reject* [30, 44, 64, 66, 82] *Endorse* [22]
A.2.iii: Other human biological material may be used improperly	*Mention* [54, 59] *Reject* [9, 66, 67, 76] *Endorse* [89]

Reasons in *Category B (chimera treatment)* focus on how the chimera will be treated once it is brought into existence, holding that either in virtue of its very existence, or owing to the conditions to which it will be subjected, the chimera will not receive a level of protection and care befitting its moral status. 23% of all coded passages (105 passages) fall in this category, making it the second-most debated category. As with the *chimera creation* category, the concerns here fall into two broad reason types, which differ on the moral status attributed to the chimera: B.1 assumes that the chimera will have a moral status akin to an animal, while B.2 imagines that a chimera might have human analogous moral status. 33% of all coded passages in this category are mentions, 27% are rejections, and 40% are endorsements (see Table 6).

**Table 6:** Mentions, rejections and endorsements of reasons concerning chimera treatment (Category B)

Reasons Concerning Chimera Treatment (Category B)	
B.1: The chimera might be violated in its animal-analogous moral status	
B.1.i: Chimera’s mere existence might be inconsistent with animal welfare and/or animal non-instrumentalization	*Mention* [9, 26] *Reject* [34, 85] *Endorse* [19, 70]
B.1.ii: Chimera’s further treatment might be inconsistent with animal welfare and/or animal non-instrumentalization	*Mention* [8, 54, 66] *Reject* [2, 4, 67, 82, 85] *Endorse* [none]
B.2: The chimera might be violated in its human-analogous moral status	
B.2.i: Chimera’s mere production might violate human-analogous respect	*Mention* [36, 49] *Reject* [34, 67, 68] *Endorse* [14, 20, 21, 53, 63]
B.2.ii: Chimera’s mere existence might be incompatible with human-analogous welfare	*Mention* [66] *Reject* [34, 68] *Endorse* [53]
B.2.iii: Chimera’s developmental options might not allow for its relevant potential^a^	*Mention* [50, 56] *Reject* [36, 68] *Endorse* [26]
B.2.iv: Chimera’s early treatment might violate human-analogous embryo protection	*Mention* [22, 41, 54] *Reject* [89] *Endorse* [39, 42, 84]
B.2.v: Chimera’s later treatment might be incompatible with human-analogous rights^b^	*Mention* [9, 26, 36, 41, 68] *Reject* [4, 8, 59, 66] *Endorse* [5, 10, 11, 19, 27, 37, 42, 56, 81, 88, 89, 92]
B.2.vi: Chimera might lack adequate human-like surrounding	*Mention* [46] *Reject* [none] *Endorse* [36, 37]
B.2.vii: Chimera might be attributed a questionable role in society^c^	*Mention* [84] *Reject* [none] *Endorse* [10, 14, 81, 82]
B.2.viii Chimera might have unclear moral status	*Mention* [21, 26, 60, 68, 71, 76, 77] *Reject* [17, 29, 38, 52, 69] *Endorse* [14, 27, 28, 32, 40, 47, 63]
B.2.ix Chimera might have human-like capacities/characteristics^d^	*Mention* [6, 17, 26, 27, 36, 61, 72, 79] *Reject* [9, 10, 50, 51] *Endorse* [11, 19, 34, 35, 89]

^a^ E.g. when a potential for rational behavior is confined to a bodily structure that will not support its development

^b^ E.g. when the chimera is experimented on without adequate consent or killed for research purposes

^c^ E.g. when the chimera is abused as an inferior member of a slave race

^d^ Insinuating that this possibility in itself constitutes an ethical problem

*Category C (chimera existence)* contains reasons concerning potential problems resulting from the existence of a chimera. This is the most heavily debated category, containing 46% of all coded passages (215 passages). Again, it contains two broad reason types: C.1 is concerned with the potential metaphysical implications of a human-animal chimera (particularly the breaking down or crossing of certain boundaries), while C.2 focuses on potential social issues (such as moral confusion or slippery slope effects). 49% of all coded passages in this category are mentions, 39% are rejections, and 12% are endorsements (see Table 7).

**Table 7:** Mentions, rejections and endorsements of reasons concerning chimera existence (Category C)

Reasons Concerning Chimera Existence (Category C)	
C.1: Crossing human-animal species boundaries could have detrimental metaphysical effects	
C.1.i: Existence of chimeras may threaten human dignity	*Mention* [2, 9, 11, 12, 20, 22, 24, 26, 27, 48, 49, 53, 73, 74, 79, 80] *Reject* [8, 34, 36, 37, 57, 60, 65–68, 71, 72, 81, 82, 85, 88] *Endorse* [14, 42, 54, 56, 63, 92]
C.1.ii: Existence of chimeras may blur species identities	*Mention* [9, 12, 20, 23, 24, 26, 36, 37, 41, 44, 45, 48, 53, 64, 70, 71, 74, 76, 88] *Reject* [4, 11, 18, 22, 46, 47, 55, 56, 66, 73, 75, 81, 85] *Endorse* [14, 17, 33, 49, 63, 80, 83, 87]
C.1.iii: Existence of chimeras may violate moral taboos^a^	*Mention* [9, 22, 26, 37, 49, 73, 88] *Reject* [11, 55, 56, 66] *Endorse* [83]
C.1.iv: Existence of chimeras may evoke instinctive repugnance^b^	*Mention* [9, 10, 12, 14, 22, 26, 34, 54, 59, 73, 93] *Reject* [13, 28, 57, 65, 66, 77, 82, 85, 88] *Endorse* [84]
C.1.v: Creation of chimeras may be unnatural	*Mention* [4, 9, 20, 22, 26, 37, 45, 48, 49, 73, 79, 80] *Reject* [11, 13, 46, 56, 66, 81, 83, 87, 88] *Endorse* [none]
C.1.vi: Creation of chimeras may amount to playing God	*Mention* [2, 20, 22, 48, 73, 87, 93] *Reject* [65, 82, 85, 88] *Endorse* [none]
C.2: Crossing human-animal species boundaries could have detrimental social effects	
C.2.i: Existence of chimeras may lead to moral confusion^c^	*Mention* [8, 9, 20, 23, 26, 27, 29, 30, 33–35, 43, 48, 49, 52, 74, 75] *Reject* [22, 24, 25, 28, 31, 38, 40, 47, 55, 58, 62, 65, 66, 78, 81, 82, 86, 87, 90, 91] *Endorse* [17, 69, 73, 93]
C.2.ii: Existence of chimeras may have slippery slope effects^d^	*Mention* [12, 22, 65, 88] *Reject* [13, 54, 82] *Endorse* [14, 32]
C.2.iii: Creation of chimeras may undermine public support for scientific research	*Mention* [22, 30, 59, 72] *Reject* [76] *Endorse* [9, 14]
C.2.iv: Creation of chimeras may result in cross-species pregnancies	*Mention* [6, 12, 23, 34, 35, 60, 61, 84] *Reject* [4, 9, 66, 67] *Endorse* [14, 54, 56]

^a^ Suggesting that these taboos demarcate essential moral borders

^b^ Suggesting that this repugnance hints to some relevant moral aberration

^c^ Supposing that the existence of chimeras leads to an erosion of important moral differences in the respective treatment of humans and animals

^d^ Supposing that the existence of chimeras, once permitted, makes it impossible to argue consistently against clear moral malpractices

Finally, *Category D (downstream effects)* is concerned with harms that may result from the application of chimera research, and the resources that must be invested in it. Only 5% of all coded passages (21 passages) fall under this category, making it the least debated group of reasons. Once more, two broad reason types can be distinguished: D.1 focuses on potential harms to individual patients, from, for example, the uncritical translation of research results or the premature transfer of material from chimeras to humans, whereas D.2 focuses on the interests of third parties, which might be impacted by the diversion of research funding, or by biosafety concerns. 52% of all coded passages in this category are mentions, 29% are rejections, and 19% are endorsements (see Table 8).

**Table 8:** Mentions, rejections and endorsements of reasons concerning downstream effects (Category D)

Reasons Concerning Downstream Effects (Category D)	
D.1: Individual medical safety might be infringed	*Mention* [4, 49, 54, 66] *Reject* [82, 85] *Endorse* [8, 19, 35]
D.2: Third party interests might be infringed	
D.2.i: Findings and substances may threaten general biosafety^a^	*Mention* [14, 26, 27, 54, 85] *Reject* [2, 72] *Endorse* [89]
D.2.ii: Funding chimera research may contradict distributive justice^b^	*Mention* [24, 89] *Reject* [13, 82] *Endorse* [none]

^a^ Particularly by spreading new diseases

^b^ Particularly by affording more financial resources than would be warranted on objective grounds

## Discussion

The frequency of endorsements, rejections and mentions of a reason cannot, on its own, lead us to a conclusion about that reason’s cogency, or about the merits of the arguments in which that reason is deployed. Nonetheless, our categorization and documentation of reasons concerning chimera research yields a descriptive account of the current debate, allowing us to highlight noteworthy trends, argumentative clusters and interesting patterns within the discussion.

### Positive reasons (category P)

#### (15% of all coded passages. Distribution within: 31% mentions, 7% rejections, 61% endorsements)

It is striking that discussions of *positive reasons* (Category P) constitute a rather small fraction of all passages retrieved (15%). Additionally, these *positive reasons* are mostly endorsed (61%) or mentioned (31%), and only rarely rejected (7%). Both phenomena can be accounted for.

The relatively low frequency of passages referring to *positive reasons* might be explained by the fact that engaging with these reasons often involves speculation concerning whether certain states of affairs will obtain. In particular, endorsing or rejecting these reasons mainly depends on scientific or medical prognosis (will chimera creation lead to advances in basic research or will it not (P.1), will chimera research foster the development of application options or will it not (P.2)?). Additionally, it is largely uncontroversial that these potential advances in basic and applied research are morally desirable and they thus do not form an attractive basis for an in-depth ethical discussion. By contrast, more intricate ethical questions concerning competition and allocation of resources are framed negatively and are thus grouped under *downstream effects* (D.2.ii). Authors of papers retrieved in a survey of ethical arguments are likely to focus on ethically controversial issues that call for discussion and analysis, while, at the same time, they may not be ideally placed to predict in a detailed manner just what benefits we might expect to obtain from chimera research. It is therefore unsurprising that these authors do not engage primarily with *positive reasons*, focusing instead on the more ethically controversial issues in the negative categories.

Concerning the relatively low rejection rate of *positive reasons*, suggestions that chimeras might contribute to basic research or could lead to valuable applications are rather vague, making targeted criticism difficult. Rejections in this field would mainly have to amount to accusations of “overselling”. This skepticism, however, is not easy to substantiate. Furthermore, it would require detailed predictions of benefits, which, as noted above, are not likely to form a central focus in the ethical literature.

### Negative reasons (categories A-D)

#### (85% of all coded passages. Distribution within: 46% mentions, 34% rejections, 20% endorsements)

The four remaining negative categories focus on more ethically controversial issues, require more ethical analysis, and involve, on the whole, less detailed empirical conjecture. This is particularly the case for the categories concerning *chimera treatment* (B) and *chimera existence* (C). For example, suggestions that bringing a chimera into existence might violate human-analogous respect (B.2.i) or that the existence of chimeras might threaten human dignity (C.1.i) requires discussion of ethical concepts (just what do human-analogous respect and human dignity amount to, and why would chimera research threaten these standards?). In addition, these discussions do not necessarily have to assume that a very specific type of chimera will exist, as, for example, any chimera with human-associated capacities or with a sufficient amount of human material might invoke concerns of human respect and human dignity (even if the existence of these types of chimeras remains, for the moment, rather visionary). To be sure, some of these issues do involve a certain degree of empirical conjecture (relatively specific capacities will be relevant to some reasons in the *chimera treatment* category (B), such as the contention that certain types of chimeras would seriously suffer (B.1.i), or that ill-treatment will result from the chimera having a human-like consciousness (B.2.ix)). Similarly, other arguments rely on certain psychological or sociological postulates (for example, reasons in the *chimera existence* category (C) assume that there are certain social taboos that the existence of chimeras might violate (C.1.iii), or that important psychological and social barriers will be threatened by the existence of chimeras, leading to moral confusion (C.2.i)). Even in these cases, however, there are hotly debated ethical concepts and issues that require sustained discussion to make a case that we should (or should not) view this as a serious ethical problem (could this suffering be justified in certain circumstances, why should a human-like consciousness be avoided, is there anything wrong with violating taboos, why is moral confusion a problem?).

It should also be noted that some of the ethical issues raised by chimera research are familiar from other bioethical contexts. This is particularly true for reasons concerning *chimera creation* (A), *chimera treatment* (B – particularly B.1, where the chimera is presumed to have animal-analogous moral status) and *downstream effects* (D), which refer to common problems of animal experimentation (A.1, B.1), the use of human biological material (A.2), safety (D.1, D.2.i), and justice (D.2.ii). It is thus unsurprising that, in a survey of academic literature, which is inherently striving for originality and innovation, these reasons are reiterated relatively infrequently (B.1, D), or merely mentioned rather than discussed in a sustained manner (A). In addition, the safety-based concerns in D.1 and D.2.i, like the *positive reasons*, are predicated on specific scenarios coming to pass (will it indeed be the case that the results of chimera research pose a threat to the individual (D.1) or to biosafety in general (D.2.i), and, if so, how significant are the risks?). Although there is a more controversial ethical issue at the heart of these concerns than within the category of *positive reasons* (which risks would be acceptable?), this question is not an attractive candidate for sustained ethical consideration, due to the fact that it is not specific to chimera research and thus tends to bypass the discussion of novel issues in favor of appeals to general moral standards concerning risk-taking.

Finally, the fact that more articles are concerned with *negative reasons* (A-D) than with *positive reasons* (P) does not imply a negative attitude towards chimera research, as reasons discussed might only be mentioned, or even ultimately rejected rather than endorsed by the author. At the same time, however, the fact that reasons in the negative categories (A-D) exhibit an overall surplus of rejections (34%) over endorsements (20%) does not indicate a positive attitude towards chimera research either, as it is possible to repudiate certain reasons against conducting chimera research without approving of the practice overall.

### Reasons concerning chimera creation (category a)

#### (11% of all coded passages. Distribution within: 58% mentions, 28% rejections, 13% endorsements)

The relatively infrequent appearance of passages within this category (11%) might be attributed to the fact that these reasons rehash well-known arguments concerning the treatment of animals in research (A.1) and the use of human biological material, including human embryos and human eggs (A.2) (see above). As such, arguments dealing with these issues can be found in existing bioethical literature, requiring only minor amendments for application to the cases at hand. There is thus a limited incentive for authors to engage in sustained discussion of reasons pertaining to *chimera creation*. Of course, this by no means precludes their practical relevance.

### Reasons concerning chimera treatment (category B)

#### (23% of all coded passages. Distribution within: 33% mentions, 27% rejections, 40% endorsements)

The *chimera treatment* category is composed of two broad reason types – one based on the assumption that chimeras will have animal-analogous moral status (B.1), while the other proceeds from the assumption that chimeras will have human-analogous moral status (B.2). The relatively high proportion of endorsements (40%) compared to rejections (27%) for both broad reason types might be a result of the fact that challenging these reasons is likely to be based on specific assumptions about the capacities of chimeras (for example, doubting that chimeras would ever attain human-analogous capacities (see B.2.ix)), which, as noted above, may not be the area of expertise of many authors writing ethical papers. It is only in very few cases that it seems possible to challenge these arguments through questioning the moral standards to which they appeal (for example, by arguing that a being with a certain potential is not necessarily owed corresponding developmental options (see B.2.iii)), but generally, the moral standards invoked are largely uncontroversial. Thus, while pointing out problems with *chimera treatment* may involve novel ethical discussion (through highlighting novel dangers of maltreatment, instrumentalization etc. in biomedical practice), the repudiation of these arguments will mostly be a matter of suggesting that these potential scenarios will not ultimately materialize.

Reasons predicated on the idea that chimeras have animal-analogous status (B.1) suffer from the familiarity of animal ethics arguments in general, which could explain their infrequent representation in comparison to reasons which involve speculation that a chimera might have human-analogous moral status (B.2). As above, this does not preclude their importance in practice, particularly as the notion of a chimera with human-analogous traits is rather speculative. Furthermore, it should be noted that concerns with animal protection are distributed between the *chimera treatment* category (B, specifically B.1) and the *chimera creation* category (A, specifically A.1), depending on whether the authors are concerned with harms to animals in the chimera generation process, or to the resulting animal-analogous chimera. Animal issues thus make up a greater proportion of the debate than may be apparent at first glance.

### Reasons concerning chimera existence (category C)

#### (46% of all coded passages. Distribution within: 49% mentions, 39% rejections, 12% endorsements)

Reasons concerning *chimera existence* make up a significant proportion of all retrieved passages (46%). One explanation for this, and particularly for the higher prevalence of discussions concerning *chimera existence* (C) compared to discussions concerning *chimera treatment* (B), is that much discussion of the latter involves scrutiny of specific types of chimeras (the origin of a chimera’s cells, or its prospective capacities, etc., are likely to be relevant factors in determining how it should be treated). Reasons concerning *chimera existence*, by contrast, mostly deal with human-animal chimeras in general, invoking the potential metaphysical or social implications of these beings’ mere presence.

The overall proportion of rejections (39%) in the *chimera existence* category is quite high compared to endorsements (12%). The particularly low frequency of endorsements of reasons C.1.iii-C.1.vi (stating that the creation of chimeras might violate moral taboos, meet with instinctive repugnance, corrupt the natural order, or amount to playing God), relative to mentions and rejections, may suggest that discussions and refutations of these reasons are, predominantly, targeted at straw men. Alternatively, these reasons could appear, or be perceived to appear, in lay discourse, rather than in scholarly debate.

The fact that reason C.2.i (chimeras might generate moral confusion) has so few endorsements and so many mentions and rejections may be an editorial artefact. The first paper to advance this reason was a target article in the American Journal of Bioethics [73] and thus was accompanied by a series of open peer commentaries, which tend to take a critical stance toward the article they address.^3^

### Reasons concerning downstream effects (category D)

#### (5% of all coded passages. Distribution within: 52% mentions, 29% rejections, 19% endorsements)

Reasons concerning *downstream effects* constitute the least debated category (5%). Due to the paucity of data, reliable trends cannot be identified. There are several possible explanations for the infrequent discussion of *downstream effects* within the debate. First, the calculation of *downstream effects* requires making concrete predictions about the results of chimera research (whether, for example, they are likely to present threats to safety). This is compounded by the fact that the concerns discussed in this category often require far-reaching forecasts of consequences in the distant future, which are even more difficult to predict. The relatively far-off nature of these potential consequences also means that they might be viewed as less immediately urgent. Finally, the safety (D.1, D.2.i) and justice-based (D.2.ii) concerns contained in *downstream effects* are not specific to chimera research, but could be invoked in any biomedical context. All of these aspects might contribute to *downstream effects* being less attractive candidates for discussion.

### Limitations

Although we devised the conception and methodology of our work with close regard to its purpose and demands, this study has certain limitations that need to be critically addressed. More precisely, these limitations concern the risks of: (1) data not being comprehensively included in our survey; (2) results not being unanimously extractable from the data; (3) conclusions not being readily inferable from the results.

### Limitations of data, due to selection criteria and search procedures

As noted above (see Methods), we restricted our review of academic literature to English sources and to articles in international peer-reviewed journals. The restriction to English literature risks overlooking arguments from other cultural spheres. However, because English has become the dominant language for international bioethical discourse, we are confident that our data accurately reflects the scholarly debate at an international level. The restriction to peer-reviewed journal articles might also lead to the inadvertent exclusion of certain arguments. However, the inclusion of non-peer-reviewed literature would make it difficult to consistently exclude lay sources, feature pages, and other public opinion position papers. In addition, reports, surveys, encyclopedia entries, handbook articles etc. often summarize existing debates, and thus may lead to a distortion of data through a double-counting of reasons. The restriction to English [95–98] and peer-reviewed journal articles [95–97, 99, 100] is common in systematic reviews of reasons.

Additionally, it is possible that not all publications conforming to our selection criteria were included, because they do not appear in the databases searched, or because our search strings did not pick them up. It is also possible that, of the articles retrieved, we failed to identify some articles that met our inclusion criteria. We attempted to mitigate the latter limitation by requiring consensus concerning inclusion.

### Limitations of results, concerning the attribution of text passages to reason types

Because coding of the passages could not be based on a simple search for keywords or catch phrases (the word “dignity”, for example, is deployed both to express concerns about the treatment of a chimera and the integrity of the human species), reasons were identified by a close reading and analysis of the texts. This introduces the danger of subjectivity, which we attempted to mitigate by coding passages independently, and eliminating disparities through discussion.

### Limitations of conclusions

As outlined above (see Discussion), the number of mentions, rejections and endorsements of specific reasons does not allow us to draw any normative conclusions about the quality of the arguments, but rather provides a purely descriptive account of the current debate. Even descriptive conclusions, however, can only be drawn with caution. As outlined in the discussion above, the frequencies of reason mentions, endorsements and rejections might often be explained as a function of the peculiarities of academic bioethical debate. In particular, it is thus possible that our results do not mirror the concerns that bioethicists (even the authors included in our review) would identify as the most pressing. For instance, a bioethicist might publish a paper on a novel issue due to its interesting implications, or to capitalize in a gap on the debate. At the same time, however, she might hold that the most urgent moral problems with chimera research are the more familiar bioethical problems (such as animal suffering or translational risk).

## Conclusion

To the best of our knowledge, this review is the first systematic review of ethical arguments concerning chimera research. We have identified five broad categories of reasons: *positive reasons* (P), and *negative reasons* pertaining to *chimera creation* (A), *chimera treatment* (B), *chimera existence* (C), and *downstream effects* (D). Within these categories, we identified 12 broad and 31 narrow types of reasons, and surveyed the frequencies of their mentions, rejections and endorsements. We hope that the classification into these five broad categories in particular provides an easily accessible overview of the debate, through supplying a systematic classification that reveals the disparate nature of the concerns advanced by various authors across different categories, as well as highlighting the connections between positions taken within the categories.^4^

As an enterprise in descriptive ethics, a systematic review of reasons, as noted above, can yield no immediate normative answers concerning where this debate should move, or which approaches are ethically superior to others. Rather, by outlining the structure of the debate, presenting and interpreting trends, and revealing prominent positions, we have attempted to provide orientation in this complex debate, thus facilitating future academic discussion and policy decisions.

However, some lessons can be drawn from our results. First, we have revealed that ethical stances towards chimera research focus on highly diverse aspects of this scientific endeavor, which invoke a variety of concerns in biomedical ethics (the expected benefits of scientific advances, the ethics of using laboratory animals and human material, the protection of higher organisms, the ontology and sociology of interspecies relations, and the responsibility for more remote research consequences). We suspect that the highly fragmented nature of this debate can undermine coherent assessment of, and ethical consensus concerning the permissibility of, proposed chimera research projects. We hope that our contribution might begin to ameliorate this: by highlighting the different categories of ethical concern, our classification system may help to allow ethicists and policy-makers to get on the same page, and reduce the risk of them talking past each other. Second, our results highlight a potentially fruitful area of further inquiry: work exploring the connections and interdependence of the concerns across the different categories [101]. Ultimately, we need a unified picture of the ethical challenges of human-animal chimera research in order to come to a more integrated assessment of this rapidly developing technology.

## Supplementary information

**Additional file 1.** Illustrative quotation for each reason type. We have included this file to demonstrate how we assigned reasons to text passages. It contains an exemplary passage for every reason, formulated as endorsement, rejection or mention. This table should help to further understand the classification system of reasons per se, but also to illuminate how mentions demarcate from rejections and/or endorsements.

## Data Availability

The authors ensure full transparency of the review process. The full search strategy is presented in Fig. 1 as well as Table 1 of the manuscript, and all databases searched have been listed in the manuscript. All data generated or analyzed during this study are included in this published article (and its supplementary information files).

## Abbreviations

MEDLINE: Medical Literature analysis and retrieval system online (a database)
MeSH: Medical subject headings
Embase: Excerpta Medica database (a database)

## Footnotes

1 “Chimera” is the most frequently used term in discussions on this topic, typically encompassing any creature arising from a mixture of human and animal material, including hybrids and cybrids.

2 This means that some prominent literature on chimera research was excluded: For example, Monika Piotrowska’s “Transferring Morality to Human-Nonhuman Chimeras” [94] was excluded due to the fact that it does not discuss reasons for or against conducting chimera research, but rather puts forward a potential classification system that might allow us to better determine how to treat human-nonhuman chimeras. By contrast, two peer commentaries on this article that do contain discussion of reasons for or against chimera research were included in our survey [21, 29].

3 It should be noted that another target article [47] focusing on moral confusion was published in the same journal in 2012 alongside five peer commentaries. Here, however, the results were not as skewed, with two endorsements [17, 69], one mention [52] and two rejections [38, 40] identified in the peer commentaries.

4 It is also important to note that the debate on chimera research is ongoing, and has particularly been fueled by recent developments in chimera research for the purposes of organ transplantation. Because of the end date of our systematic review, some more recent, significant contributions to the debate have not been included [101–112].
