# Supplementary material for: Ethical arguments concerning human-animal chimera research: a systematic review
Source: BMC Med Ethics. 2020 Mar 23;21:24. doi: 10.1186/s12910-020-00465-7 (PMC7092670; doi:10.1186/s12910-020-00465-7)
Supplement: Supplementary file 1 — Additional file 1. Illustrative quotation for each reason type. We have included this file to demonstrate how we assigned reasons to text passages. It contains an exemplary passage for every reason, formulated as endorsement, rejection or mention. This table should help to further understand the classification system of reasons per se, but also to illuminate how mentions demarcate from rejections and/or endorsements. [file 12910_2020_465_MOESM1_ESM.docx]

**Additional File 1: Illustrative Quotation for Each Reason Type**

| **Category P: Positive Reasons** | |
| --- | --- |
| P.1: The creation of chimeras may advance basic research | **Endorsement:** *From the point of view of more basic research, cybrids would provide a new model for studying nuclear cytoplasmic interactions, nucleus reprogramming and cell differentiation during embryo development.* [1] |
| P.2: The creation of chimeras may produce benefits for humans | |
| P.2.i: New therapies might be developed on the basis of chimera research | **Endorsement:** *We believe that use of animal eggs in the creation of cytoplasmic hybrid embryos will help to overcome the current shortage of human eggs available for research and that use of animal eggs is required to enable researchers to develop the practical techniques which may be required for eventual production of cell based therapy through this method using human eggs*. [2] |
| P.2.ii: Chimeras might serve as sources of transplantable organs and tissues | **Endorsement:** *The most obvious advantage of chimaera organs is that they would provide an inexhaustible source of organs for transplantation. Anyone in need of a new organ could provide iPSCs, which would be inserted in a pig embryo prior to implantation and gestation. After around 6 months, the resulting pig would be sacrificed and the human organ removed and implanted in the original donor. Hundreds of thousands of lives would be saved every year if this technique was widely adopted […].* [3] |
| P.2.iii: Chimera research might open ways to human enhancement | **Endorsement:** *Suppose that we were to find that animals that have a significantly longer lifespan than human beings, such as turtles, contained genetic sequences that reduced the rate of telomere degradation. It might then be possible to transfer these sequences into the human genome, radically prolonging life or compressing aging. This would be enhancement for radically longer life or less aging […].* [4] |
| P.3: The creation of chimeras may prevent direct harm to humans or animals | **Endorsement:** *Third, a/h chimeras may serve as an alternative source for human embryonic stem cells (hESCs). Because the harvesting of hESCs from a chimeric embryo may not involve the destruction of a human embryo, but rather that of an animal embryo which contains hESCs, the ethical quandaries surrounding the use of human embryos to derive hESCs would be circumvented.* [5] |
| P.4: The creation of chimeras may have other benefits | **Mention**: *Alternatively, it might be argued that the challenge is less to avoid a direct ethical ill and more to understand the mental capacities of engrafted animals and to treat them in a manner appropriate to their moral status. Indeed, it might even be argued that such changes constitute a potential benefit to*  *the engrafted animal, insofar as the changes are viewed as enhancements of the sort we value for ourselves.* [6] |
| **Category A: Reasons regarding the process resulting in a chimera** | |
| A.1: Animals might be mistreated | |
| A.1.i: General animal welfare may be infringed | **Mention:** *A further ethical objection to research involving crossing species boundaries highlights the wrong that such research visits on nonhuman animals.* [7] |
| A.1.ii: Special protection of higher animals such as primates may be infringed | **Endorsement:** *Great Apes should not be used in research unless (1) their participation is realistically expected to pose no more than minimal risk to them or (2) greater risks are justified by the prospect of direct veterinary benefit to them and the absence of alternatives offering a better benefit/risk ratio. Moreover, if Great Apes find participation aversive, making clear signs that they don’t want to continue, these communications should count as dissent and should disqualify the subjects from further participation unless they face substantial veterinary need and participation in the study is the best hope for meeting it.* [8] |
| A.2: Human beings/human material might be mistreated/misused | |
| A.2.i: Human embryo protection may be neglected | **Mention:** *[…] the use of human embryos to generate stem cell material, in the process destroying the embryo, has clashed with values that accord a high moral status to the embryo […]. In this respect, hESC science forms part of the wider and continuing debate over biomedical technologies that act on the embryo, such as assisted reproductive technologies, preimplantation genetic diagnosis, and abortion with its focus on the later stage of development of the fetus.* [9] |
| A.2.ii: Undue forms of human egg donation may occur | **Rejection:** *[…] even if researchers eventually need additional quantities of human-generated human eggs, this is not necessarily bad overall, despite the possible dangers. The harms of egg donation in such circumstances should be weighed against the good that could be done—supposing, of course, that such donations are made with proper informed consent, and not under duress or undue influence.* [10] |
| A.2.iii: Other human biological material may be used improperly | **Rejection:** *Whereas insufficient respect for the human origin of the cells may begin to capture some of people’s reasons for being upset […], this issue appears to me to be bad ethics, or nonsense, masquerading as ethics. What is evidence for “lack of respect” besides the use—the very point at issue? And, assuming consent is obtained, is it not up to the acknowledged source of the tissue to decide what is a “respectful” use? Like all such appeals—including what I consider to be vacuous appeals to “human dignity”—it is difficult to know when (if ever) an aesthetic aversion or a theological one constitutes a genuine ethical issue.* [11] |
| **Category B: Reasons regarding the treatment of a created chimera** | |
| B.1: The chimera might be violated in its animal-analogous moral status | |
| B.1.i: Chimera’s mere existence might be inconsistent with animal welfare and/or animal non-instrumentalization | **Rejection:** *Given proper raising, handling, and other care techniques, there is good reason to believe that the animals will have such an existence, albeit shorter than some might want. There are those who might even label the human-animal chimera lives as flourishing up to the moment of death; thus, chimeras created for death are not injured inherently by their mere existence or why they came into being.* [12] |
| B.1.ii: Chimera’s further treatment might be inconsistent with animal welfare and/or animal non-instrumentalisation | **Rejection:** *Also, it is uncontroversial that saving people’s lives, or ameliorating people’s great suffering, are aims that justify harming or killing certain sentient animals, when there are not other means available to us. Therefore, if the research aims for using chimeras are geared towards saving people’s lives, or ameliorating people’s great suffering, then creating them […] is morally justifiable.* [13] |
| B.2: The chimera might be violated in its human-analogous moral status | |
| B.2.i: Chimera’s mere production might violate human-analogous respect | **Rejection:** *“[…] creating a chimera […] would create a being with human dignity. Therefore, it may be claimed that given this, and the belief that creatures in possession of a dignity ought not be used merely as a means to an end, chimeras […] should not be created”.* [14] |
| B.2.ii: Chimera’s mere existence might be incompatible with human-analogous welfare | **Endorsement:** *Intentionally creating compromised human beings or part-human beings is cruel to the creature created (it is, for example, […], able to exercise only compromised human facilities, […], and perhaps not able to fend for itself).* [15] |
| B.2.iii: Chimera’s developmental options might not allow for its relevant potential | **Mention:** *Proponents of an argument from human dignity would maintain that to create a human-nonhuman chimera would either diminish or wholly eliminate the possibility that humans could exercise the cluster of capacities and characteristics that are associated with human dignity, treating them solely as a means to others’ ends.* *By giving nonhumans some of the physical components necessary for development of the capacities associated with human dignity, and encasing these components in a nonhuman body where they would either not be able to function at all or function only to a highly diminished degree, those who would create human-nonhuman chimeras would denigrate human dignity.* [16] |
| B.2.iv: Chimera’s early treatment might violate human-analogous embryo protection | **Endorsement:** *In addition, the embryos in these experiments were created with the intention that they would later be killed, to obtain their stem cells or to preclude further growth, and if there is a chance that these embryos are human at all, it is wrong to kill them.* [17] |
| B.2.v: Chimera’s later treatment might be incompatible with human-analogous rights | **Endorsement:** *If higher consciousness did emerge—and we knew about it—then there would be significant moral questions to be asked: not least that it is equally possible that, like any rational being, it will resent a confined existence that is absent of basic rights; and these are the same rights that normally preclude nonconsensual experimentation in human beings.* [18] |
| B.2.vi: Chimera might lack adequate human-like surrounding | **Endorsement:** *But the lack of flourishing also could result from the fact that the chimeras produced are unlikely to live in a social context that would allow them to develop and function according to the complex capacities that they might have. Thus, even if they were not used for experimental purposes, it is highly improbable that society would use resources to ensure that such creatures develop to the fullest extent of their capacities.* [19] |
| B.2.vii: Chimera might be attributed a questionable role in society | **Endorsement:** *Researchers might support the development of a chimera for economic or scientific reasons—such a creature could attract more attention and funding than a circus stunt. Such an interest—which regards the creature as a means rather than also an end-in-itself—would be obviously unethical and probably self-defeating.* [20] |
| B.2.viii Chimera might have unclear moral status | **Endorsement:** *What critics can demand, and proponents must answer, is that this uncertainty of moral status be balanced against the possible risk of moral harm that may be done to those created individuals. This reframes the debate into one of how much that uncertainty or moral risk may be mitigated, given other facts about which we have more solid empirical or theoretical grounding.* [21] |
| B.2.ix Chimera might have human-like capacities/characteristics | **Endorsement:** *Several examples of chimeric experiments highlight which studies would be ethically unacceptable. During early development of sheep/goat blastocyst chimeras, increasing the proportion of transplanted cells in the inner cell*  *mass can bias donor- or host-specific characteristics. If human cells greatly out-numbered host cells in an early blastocyst of a developmentally similar host […], it is conceivable that human-like psychological capacities might emerge in the resulting chimera.* [22] |
| **Category C: Arguments regarding the presence of chimeras** | |
| C.1: Crossing human-animal species boundaries could have detrimental metaphysical effects | |
| C.1.i: Existence of chimeras may threaten human dignity | **Rejection:** *Imagine that, incredibly, several living members of Homo floresiensis or another hominid species were discovered on an island; they would be borderline or paradigm persons. There is no intelligible reason for thinking this discovery would threaten the moral status of Homo sapiens persons any more than the constant increase in our species’ population threatens our dignity. So the transformation of a rodent into a more personlike chimera or, more realistically, a Great Ape into a more humanlike person would not threaten human dignity.* [8] |
| C.1.ii: Existence of chimeras may blur species identities | **Rejection:** *Therefore our moral unease about chimeras might well be related not only to the fragile (and many would argue indefensible) line that we often draw between human and nonhuman animals, but more generally to the growing recognition of the very fragility of scientific categories themselves, as they are affected by technological and theoretical developments, the changing goals and context of scientific research, and social negotiation within the scientific community.* [23] |
| C.1.iii: Existence of chimeras may violate moral taboos | **Mention:** *For others, the inherent revulsion is grounded in concerns about the anticipated violation of well-entrenched (although perhaps somewhat inchoate) taboos about the mixing of humans and nonhumans.* [7] |
| C.1.iv: Existence of chimeras may evoke instinctive repugnance | **Mention:** *Some people may* feel *an instinctive moral repugnance towards the prospect of creating cybrids. However, precisely because this is not a rational position, but a position based upon an instinctive, visceral reaction to something thought of as deserving disgust, it is very difficult to deal with it in a rational discussion. We are not suggesting here that such reactions are completely meaningless for the formations of our moral judgments. On the contrary, they could be an emotional starting point, which, nevertheless, should be elaborated and supported with rational arguments in order to construct a moral judgment […].* [1] |
| C.1.v: Creation of chimeras may be unnatural | **Rejection:** *The “unnaturalness” argument makes assumptions about the interpretation of biological*  *phenomena and the elucidation of ethical values that does not, and could not, follow from what we have learned of the evolution and development of humans and nonhumans. Consequently, we set aside the “unnaturalness” objection to the creation of human-nonhuman chimeras on grounds that it equates, and thereby confuses, biological description with the justification of ethical norms. It therefore provides insufficient warrant for judging the creation of human-nonhuman chimeras to be wrong.* [16] |
| C.1.vi: Creation of chimeras may amount to playing God | **Mention:** *According to some, crossing species boundaries is about human beings playing God and in so doing challenging the very existence of God as infallible, al-powerful, and all-knowing.* [24] |
| C.2: Crossing human-animal species boundaries could have detrimental social effects | |
| C.2.i: Existence of chimeras may lead to moral confusion | **Rejection:** *First, it is not at all obvious why membership in a species (as opposed to possession of properties that members of that species commonly have) should entail radically different moral status. Second, if it were, then we would be much less likely to be confused by human-nonhuman chimeras, because we would be able to say in what respect a chimera had to be similar to us to warrant the treatment we think appropriate to human beings. If this is right, then we find chimeras confusing because our views of the moral status of human and nonhuman animals are not adequate as they stand, in which case our confusion is not a reason not to create them.* [25] |
| C.2.ii: Existence of chimeras may have slippery slope effects | **Mention:** *According to one such argument, the creation of humanesque cytoplasmic hybrid embryos will inevitably lead to the creation of other ethically objectionable human/nonhuman animal embryos, such as true hybrid embryos (“embryos which are created by mixing human sperm and animal eggs or human eggs and animal sperm”) and human chimera embryos.* [7] |
| C.2.iii: Creation of chimeras may undermine public support for scientific research | **Mention:** *Given the combination of concerns about interspecies mixing and the creation of research embryos, an argument can be made that cybrid creation […] could risk the loss of public support for the field of regenerative medicine.* [26] |
| C.2.iv: Creation of chimeras may result in cross-species pregnancies | **Mention:** *[…] the introduction of human gonad-like organoids into animal models might raise concerns about the possibility of inadvertent cross-species fertilization involving human and non-human gametes.* [27] |
| **Category D: Reasons regarding the generation and application of results from chimera research and of chimeric material** | |
| D.1: Individual medical safety might be infringed | **Mention:** *Though human beings are, generally, the beneficiaries of research into admixed organisms, some human beings may be harmed by these developments. All medical developments must be tested on human beings, and the first recipients of nonhuman or transgenic material may incur a significant risk.* [28] |
| D.2: Third party interests might be infringed | |
| D.2.i: Findings and substances may threaten general biosafety | **Mention:** *This issue of human-nonhuman combinations raises a number of safety concerns, in particular the danger of allowing diseases to cross the species barrier, as happened with Creutzfeldt-Jacob disease, HIV and various influenza epidemics.* [29] |
| D.2.ii: Funding chimera research may contradict distributive justice | **Rejection:** *Finally, others could point out that economical resources dedicated to the research on cybrids, whose therapeutic effects would most likely regard only the elite, could be better allocated if they were addressed to world pandemics, such as AIDS. A consistent application of this argument would require us stop the research on the causes of, and the therapies for, the very rare diseases. Should we really stop researching them?* [1] |

**REFERENCES**

1. Camporesi S, Boniolo G. Fearing a non-existing Minotaur? The ethical challenges of research on cytoplasmic hybrid embryos. J Med Ethics. 2008;34(11):821-5.

2. Bahadur G, Iqbal M, Malik S, Sanyal A, Wafa R, Noble R. Admixed human embryos and stem cells: legislative, ethical and scientific advances. Reprod Biomed Online. 2008;17 (Suppl 1):25-32.

3. Shaw D, Dondorp W, de Wert G. Using non-human primates to benefit humans: research and organ transplantation. Medicine, Health Care and Philosophy. 2014;17(4):573-578.

4. Savulescu J. Human-animal transgenesis and chimeras might be an expression of our humanity. Am J Bioeth. 2003;3(3):22-5.

5. Eberl JT, Ballard RA. Exercising restraint in the creation of animal-human chimeras. Am J Bioeth. 2008;8(6):45-6.

6. Greene M, Schill K, Takahashi S, Bateman-House A, Beauchamp T, Bok H, et al. Ethics: Moral issues of human-non-human primate neural grafting. Science. 2005;309(5733):385-6.

7. Baylis F. Animal eggs for stem cell research: a path not worth taking. Am J Bioeth. 2008;8(12):18-32.

8. deGrazia D. Human-animal chimeras: human dignity, moral status, and species prejudice. Metaphilosophy. 2007;38(2-3):310-329.

9. Salter B, Harvey A. Creating problems in the governance of science: Bioethics and human/animal chimeras. Science and Public Policy. 2014;41(5):685-696.

10. Palacios-Gonzalez C. Ethical aspects of creating human-nonhuman chimeras capable of human gamete production and human pregnancy. Monash Bioeth Rev. 2015;33(2-3):181-202.

11. Rollin BE. Of mice and men. Am J Bioeth. 2007;7(5):55-7.

12. Cooley DR. Genetically Engineering Human-Animal Chimeras and Lives Worth Living. Between The Species. 2008;81-19.

13. Palacios-Gonzalez C. Chimeras intended for human gamete production: an ethical alternative? Reprod Biomed Online. 2017;35(4):387-390.

14. Palacios-González C. Human dignity and the creation of human–nonhuman chimeras. Medicine, Health Care and Philosophy. 2015;18(4):487-499.

15. Johnston J, Eliot C. Chimeras and "human dignity". Am J Bioeth. 2003;3(3):W6-w8.

16. Karpowicz P, Cohen CB, van der Kooy D. Developing human-nonhuman chimeras in human stem cell research: ethical issues and boundaries. Kennedy Inst Ethics J. 2005;15(2):107-34.

17. Seyfer TL. An overview of chimeras and hybrids. Natl Cathol Bioeth Q. 2006;6(1):37-49.

18. Capps B. Do Chimeras Have Minds? Camb Q Healthc Ethics. 2017;26(4):577-591.

19. de Melo-Martin I. Chimeras and human dignity. Kennedy Inst Ethics J. 2008;18(4):331-46.

20. Sagoff M. Further thoughts about the human neuron mouse. Am J Bioeth. 2007;7(5):51-2.

21. Haber MH, Benham B. Reframing the ethical issues in part-human animal research: the unbearable ontology of inexorable moral confusion. Am J Bioeth. 2012;12(9):17-25.

22. Karpowicz P, Cohen CB, van der Kooy D. It is ethical to transplant human stem cells into nonhuman embryos. Nat Med. 2004;10(4):331-5.

23. Ankeny RA. No real categories, only chimeras and illusions: the interplay between morality and science in debates over embryonic chimeras. Am J Bioeth. 2003;3(3):31-3.

24. Robert JS, Baylis F. Crossing species boundaries. Am J Bioeth. 2003;3(3):1-13.

25. Bok H. What's wrong with confusion? Am J Bioeth. 2003;3(3):25-6.

26. Chapman A, Hiskes AL. Unscrambling the eggs: cybrid research through an Embryonic Stem Cell Research Oversight Committee (ESCRO) lens. Am J Bioeth. 2008;8(12):44-6.

27. Munsie M, Hyun I, Sugarman J. Ethical issues in human organoid and gastruloid research. Development. 2017;144(6):942-945.

28. Jones DA. The ethics of creating chimeras and other admixed organisms. Ethics and Medicine. 2012;28(3):81-93.

29. Jones DA. Is the creation of admixed embryos "an offense against human dignity"? Hum Reprod Genet Ethics. 2010;16(1):87-114.
